# Anticoagulation delay does not affect the functional outcome of cerebral venous thrombosis

**DOI:** 10.18632/aging.103353

**Published:** 2020-06-18

**Authors:** Kangxiang Ji, Longfei Wu, Wenbo Zhao, Chuanjie Wu, Yaoming Xu, Jiangang Duan, Ran Meng, Feng Yan, Jian Chen, Di Wu, Yinghao Luo, Xunming Ji

**Affiliations:** 1 China-America Institute of Neuroscience, Xuanwu Hospital, Capital Medical University, Beijing 100053, China; 2 Department of Neurology, Xuanwu Hospital, Capital Medical University, Beijing 100053, China; 3 Department of Neurosurgery, Xuanwu Hospital, Capital Medical University, Beijing 100053, China

**Keywords:** sinus thrombosis, anticoagulation delay, outcome, headache

## Abstract

Available knowledge about the impact of anticoagulation delay on outcomes of patients with cerebral venous thrombosis (CVT) is limited. We therefore assessed the factors influencing anticoagulation delay and investigated the effect of this delay on outcomes of CVT patients. Anticoagulation delay was defined as the time interval between symptom onset and anticoagulation initiation. The primary outcome was a modified Rankin Scale (mRS) score > 2 at the final follow-up. A total of 164 eligible patients were included. The median anticoagulation delay was 9 days. Cerebral hemorrhage on admission neuroimaging correlated with earlier anticoagulation (p = 0.040). Anticoagulation delay was not associated with poor functional outcome (mRS > 2), but it was associated with residual headache across the entire cohort (earlier anticoagulation: 15/76 [19.7%] vs. later anticoagulation: 28/79 [35.4%]; p = 0.029) and in the subgroup with isolated intracranial hypertension (earlier anticoagulation: 4/25 [16.0%] vs. later anticoagulation: 14/27 [51.9%]; p = 0.007). Anticoagulation delay was found to be common among patients with CVT. Anticoagulation delay was not associated with poor functional outcome, but may have led to an increased risk of residual headache across our entire cohort and in the subgroup with isolated intracranial hypertension.

## INTRODUCTION

Cerebral venous thrombosis (CVT) is a unique type of cerebrovascular disease that varies greatly in its clinical course and prognosis, from complete recovery to disability or death [1, 2]. CVT is usually treated with anticoagulant therapy. Timely and effective anticoagulant therapy can prevent thrombus growth, promote thrombus regression, and improve patient outcome. Conversely, a delay in starting anticoagulation may allow progressive thrombosis, a worsening of clinical status, a more complicated condition, and/or an increased thrombus burden, all of which are detrimental to the outcomes of CVT patients. Unfortunately, the diverse clinical manifestations and neuroimaging features of CVT often lead to delays in admission and diagnosis, which in turn lead to delays in anticoagulation.

Delay in admission and diagnosis in CVT has been reported in previous studies [3, 4], but each of them is not equivalent to anticoagulation delay. First, it may take several days from admission to confirmed diagnosis or anticoagulation, during which time clinical worsening may occur [5, 6]. Second, even after diagnosis, some patients are not treated with anticoagulant therapy in the above studies. Thus, clinical outcomes may be confounded by a lack of uniform therapy. Additionally, the effect of anticoagulation delay on residual headache, as one of the most common sequelae specific to CVT, has not been evaluated.

In the present study, therefore, we sought to identify the potential variables that influence anticoagulation delay and to analyze the effects of this delay on outcomes in a large single-center cohort of patients with CVT. Of particular interest was the effect of anticoagulation delay on residual headache.

## RESULTS

Between January 2014 and July 2018, a total of 179 CVT patients were enrolled in the registry. Among them, 5 patients were excluded because of our inability to determine the day of onset due to an insidious clinical course and a history of chronic headache, while 10 patients less than 14 years old were also excluded. Therefore, the remaining 164 eligible patients who were treated initially with heparin and had valid information on anticoagulation delay were included in the present study. The median age of these patients was 35 years (interquartile range [IQR], 25-49), and 53% were female. The initial anticoagulation treatment was subcutaneous injection of low-molecular-weight heparin (LMWH) in 134 patients and intravenous unfractionated heparin (UFH) in 30 patients.

### Anticoagulation delay

The median anticoagulation delay was 9 days (IQR, 4-16). Earlier anticoagulation was associated with cerebral hemorrhage on admission neuroimaging (32/80 [40.0%] vs. 21/84 (25.0%); p = 0.040). Coma (Glasgow Coma Scale [GCS] score < 9) appeared to be more frequent in patients with earlier anticoagulation than later anticoagulation, but the difference did not reach statistical significance (12/80 [15.0%] vs 5/84 [6.0%]; p = 0.057). There were no significant differences between patients with earlier anticoagulation and later anticoagulation with respect to gender, age, headache, papilledema, isolated intracranial hypertension (IIH) on admission, seizure, motor deficit, aphasia, mental disturbance, any parenchymal lesion, edema/infarct on admission neuroimaging, isolated cortical vein thrombosis, cancer, and central nervous system infections. Additional details are presented in Table 1.

**Table 1:** Association between anticoagulation delay and baseline variables.

**Baseline variables**	**Earlier anticoagulation (N=80)**	**Later anticoagulation (N=84)**	**p value**
Female, n (%)	41/80 (51.3)	46/84 (54.8)	0.652
Age, median (IQR), y	33.5 (24, 49)	36.5 (27, 49)	0.226
Symptoms and signs			
Headache, n (%)	74/80 (92.5)	76/84 (90.5)	0.643
Papilledema, n (%)	36/54 (66.7)	47/63 (74.6)	0.346
IIH on admission, n (%)	25/69 (36.2)	28/77 (36.4)	0.987
Seizure, n (%)	36/80 (45.0)	32/84 (38.1)	0.370
Motor deficit, n (%)	32/80 (40.0)	28/84 (33.3)	0.376
Aphasia, n (%)	18/80 (22.5)	18/84 (21.4)	0.868
Mental disturbance, n (%)	18/80 (22.5)	15/84 (17.9)	0.459
Coma, n (%)	12/80 (15.0)	5/84 (6.0)	0.057
Parenchymal lesions on admission neuroimaging			
Any parenchymal lesion, n (%)	52/80 (65.0)	52/84 (61.9)	0.681
Edema/infarct, n (%)	50/80 (62.5)	51/84 (60.7)	0.814
Cerebral hemorrhage, n (%)	32/80 (40.0)	21/84 (25.0)	0.040
Isolated cortical vein thrombosis, n (%)	3/80 (3.8)	3/84 (3.6)	0.951
Risk factors			
Cancer, n (%)	4/80 (5.0)	1/84 (1.2)	0.202
CNS infection, n (%)	5/80 (6.3)	9/84 (10.7)	0.306

Abbreviations: IQR, interquartile range; IIH, isolated intracranial hypertension; CNS, central nervous system.

### Association between anticoagulation delay and outcomes

The median follow-up period was 25 months (IQR, 13-42) in patients receiving earlier anticoagulation and 22 months (IQR, 15-29) in those receiving later anticoagulation. There was no significant difference in the follow-up period between the two groups (p = 0.167). The results of a multivariable logistic regression analysis of the primary outcome are summarized in Table 2. Coma (OR, 8.019; 95%CI, 1.437-44.754; p = 0.018) was the only predictor of poor functional outcome (modified Rankin Scale [mRS] score > 2). Other factors, including the anticoagulation delay (OR, 1.270; 95%CI, 0.412-3.915; p = 0.477), were not independently associated with the primary outcome.

**Table 2:** Association between anticoagulation delay and the primary outcome (multivariable regression analysis with mRS > 2 across the entire cohort).

	**OR**	**95%CI**	**p value**
Gender	0.462	0.156-1.373	0.165
Age	1.025	0.990-1.061	0.167
Cancer	1.908	0.165-22.044	0.605
CNS infection	2.897	0.616-13.628	0.178
Mental disturbance	0.864	0.198-3.770	0.846
Coma	8.019	1.437-44.754	0.018
Cerebral hemorrhage	2.971	0.895-9.868	0.075
Deep venous system thrombosis	2.476	0.642-9.549	0.188
Anticoagulation delay	1.270	0.412-3.915	0.477

Abbreviations: mRS, modified Rankin Scale; OR, odds ratio; CI, confidence interval; CNS, central nervous system.

The relationship between anticoagulation delay and outcomes across the entire cohort are shown in Table 3. Persistent residual headache was significantly more frequent in patients receiving later anticoagulation (28/79 [35.4%] vs 15/76 [19.7%]; p = 0.029). No significant differences were found between the two groups for the primary outcome or secondary outcomes (visual deficit, late seizure, or neurologic deficit).

**Table 3:** Anticoagulation delay and outcomes.

**Outcomes at the final follow-up**	**Earlier anticoagulation (N=80)**	**Later anticoagulation (N=84)**	**p value**
Primary outcome			
mRS > 2, n (%)	12/80 (15.0)	11/84 (13.1)	0.788
Secondary outcomes			
Residual headache, n (%)	15/76 (19.7)	28/79 (35.4)	0.029
Visual deficit, n (%)	12/76 (15.8)	19/79 (24.1)	0.199
Late seizure, n (%)	4/76 (5.3)	3/79 (3.8)	0.716
Neurologic deficit, n (%)	13/76 (17.1)	13/79 (16.5)	0.914

Abbreviations: mRS, modified Rankin Scale.

### Subgroup analysis

In the subgroup exhibiting isolated intracranial hypertension on admission (53 patients), the rate of residual headache was significantly higher in the later than the earlier anticoagulation group (14/27 [51.9%] vs. 4/25 [16.0%]; p = 0.007). No differences were found in other outcomes between these two groups. In the subgroup exhibiting any parenchymal lesion on admission (104 patients) and the subgroup with coma at diagnosis (17 patients), no between-group differences were detected for the primary and secondary outcomes. Additional details are presented in Table 4.

**Table 4:** Anticoagulation delay and outcomes: subgroup analysis.

**Subgroup of IIH on admission**	**Earlier anticoagulation (N=25)**	**Later anticoagulation (N=28)**	**p value**
Primary outcome			
mRS > 2, n (%)	0/25 (0)	2/28 (7.1)	0.492
Secondary outcomes			
Residual headache, n (%)	4/25 (16.0)	14/27 (51.9)	0.007
Visual deficit, n (%)	5/25 (20.0)	9/27 (33.3)	0.279
Late seizure, n (%)	0	0	-
Neurologic deficit, n (%)	0	0	-
**Subgroup with parenchymal lesion on admission**	**Earlier anticoagulation (N=52)**	**Later anticoagulation (N=52)**	**p value**
Primary outcome			
mRS > 2, n (%)	11/52 (21.2)	7/52 (13.5)	0.300
Secondary outcomes			
Residual headache, n (%)	9/48 (18.8)	13/49 (26.5)	0.360
Visual deficit, n (%)	6/48 (12.5)	9/49 (18.4)	0.424
Late seizure, n (%)	4/48 (8.3)	3/49 (6.1)	0.715
Neurologic deficit, n (%)	13/48 (27.1)	12/49 (24.5)	0.770
**Subgroup with coma at diagnosis**	**Earlier anticoagulation (N=12)**	**Later anticoagulation (N=5)**	**p value**
Primary outcome			
mRS > 2, n (%)	5/12 (41.7)	2/5 (40.0)	1.000
Secondary outcomes			
Residual headache, n (%)	2/9 (22.2)	1/5 (20.0)	1.000
Visual deficit, n (%)	3/9 (33.3)	0/5 (0)	0.258
Late seizure, n (%)	1/9 (11.1)	1/5 (20.0)	1.000
Neurologic deficit, n (%)	4/9 (44.4)	3/5 (60.0)	1.000

Abbreviations: IIH, isolated intracranial hypertension; mRS, modified Rankin Scale.

## DISCUSSION

In the present study, we systematically investigated the impact of anticoagulation delay on outcomes of patients with CVT. Cerebral hemorrhage on admission neuroimaging was identified as predictive of earlier anticoagulation. Anticoagulation delay had no significant effect on long-term functional outcomes in CVT patients. However, the anticoagulation delay increased the risk of persistent residual headache in both the entire cohort and the subgroup with isolated intracranial hypertension.

Cerebral hemorrhage was the baseline variable identified in our study that was associated with earlier anticoagulation. This result was consistent with the earlier observation that patients with more severe clinical presentation tended to be admitted to the hospital sooner and treated more actively, resulting in earlier diagnosis [4]. According to current guidelines, acute CVT should be treated immediately with initial heparin therapy, regardless of pre-treatment intracranial hemorrhage [1, 7]. It can therefore be reasonably assumed that admission or diagnosis delay is the direct cause of anticoagulation delay. Isolated intracranial hypertension syndrome, which was associated with admission or diagnosis delay in previous studies [3, 4], was not identified as predictive of anticoagulation delay in our study. This discrepancy may reflect the different distribution of diagnoses or anticoagulation delays among our cohort (the median diagnosis delay was 9 days [data not shown]). In our sample, nearly 50% of patients with seizure or any parenchymal lesion were diagnosed later (over 9 days), leading to a longer median delay in the entire cohort, whereas the median diagnosis delay in the abovementioned earlier study was only 7 days [4].

Anticoagulation delay had no significant impact on long-term functional outcomes of CVT patients because those with severe clinical manifestations tended to be admitted earlier and thus diagnosed and treated earlier. By contrast, patients with a relatively mild presentation (i.e., isolated intracranial hypertension syndrome or isolated headache) were always admitted later [3, 8]. Consequently, the benefits of earlier anticoagulation, if any, were difficult to assess across the entire CVT cohort. However, our results show that anticoagulation delay significantly increased the incidence of residual headache after CVT. The incidence of residual headache during the follow-up period varied in previous studies [9–13]. In a report from the Cerebral Venous Thrombosis Portuguese Collaborative Study Group, the incidences of any residual headache and severe headache after CVT were 47% and 8%, respectively [9]. In the International Study on Cerebral Venous and Dural Sinuses Thrombosis cohort, 14% of patients with CVT had severe headache at the end of the follow-up [10]. In a recent analysis of 161 CVT patients from a single center, 20% of survivors reported remaining frequent headaches [12]. In our cohort, 28% of CVT survivors experienced frequent or severe headaches. These incidences are difficult to compare due to the different definitions of remaining headaches. The cause of residual headaches after CVT is unclear, though remaining sinus occlusion, post-thrombotic sinus stenosis, or persistent intracranial hypertension could be the reason for residual headaches in some cases [14].

One limitation of the present study is that valuable information on the cause of residual headaches after CVT, such as persistent raised intracranial pressure or occluded sinuses, was not analyzed and warrants further study. We therefore cannot draw a conclusion about the mechanisms by which anticoagulation delay may lead to more frequent or severe headaches. Another limitation of the study is the difficulty to ascertain the exact day of onset in some of cases with a gradual clinical course or a history of chronic headache. Additionally, given the nature of our single-center design, the generalizability of our results may be hindered. Nonetheless, the strength of our study is the large sample and uniform treatment approach, which minimized outcome confusion from various treatments.

Our findings have several implications for practice. It may be appropriate to pay more attention to remaining headaches in CVT patients who received anticoagulation later. Routine follow-up imaging of the cerebral venous system to re-evaluate potential causes of the headaches may be a reasonable approach for patients with remaining frequent or severe headaches.

In conclusion, anticoagulation delay was found to be common among patients with CVT. Anticoagulation delay was not associated with poor functional outcome, but may have led to an increased risk of residual headaches across our entire cohort and in the subgroup with isolated intracranial hypertension.

## MATERIALS AND METHODS

### Study participants, baseline variables and ethics statement

The present study was based on a prospective CVT registry in our center. We reviewed electronic medical records of all patients enrolled in this registry between January 2014 and July 2018. Diagnosis of CVT was confirmed by magnetic resonance imaging combined with magnetic resonance venography, contrast-enhanced magnetic resonance venography, or digital subtraction angiography. Patients with a first episode of CVT were included if they were initially treated with heparin and had valid information regarding anticoagulation delay. CVTs in the pediatric population (less than 14 years old) were excluded. This study was approved by the Ethics Committee of Xuanwu Hospital of Capital Medical University and was carried out in compliance with the Helsinki Declaration. Written informed consent was obtained from all patients or their legal authorized representatives.

We recorded the following information: age, gender, risk factors, the day of symptom onset, the day of hospital admission, the day of confirmed diagnosis, the day of anticoagulation initiation, signs and symptoms from onset to diagnosis, isolated intracranial hypertension on admission (any combination of headache, vomiting, and papilledema with/without visual loss or sixth nerve paresis, without other neurological signs or symptoms), GCS score at diagnosis, sinuses or veins involved, characteristics of parenchymal lesions on admission neuroimaging (edema/infarct, cerebral hemorrhage).

Anticoagulation delay was defined as the time interval between symptom onset and anticoagulation initiation. Earlier anticoagulation was defined as an anticoagulation delay of less than the median for the sample. We compared potential explanatory variables between patients in the earlier anticoagulation group and those in later anticoagulation group.

### Treatment protocols

In our center, patients with CVT receive standard medical treatment according to the current guidelines immediately after diagnosis [1]. Intravenous UFH or subcutaneous LMWH in adjusted doses are options for acute CVT, though the latter is usually preferred because of its proven superior safety profile [15]. Heparin for acute CVT is usually maintained 10 to 14 days. If the patient is then clinically stable, heparin is followed by oral anticoagulants for 3 to 6 months. The duration of oral anticoagulants depends on prothrombotic conditions and patient preference.

### The primary and secondary outcomes

Follow-up visits were made at 6 and 12 months and yearly thereafter. The primary outcome was death or dependency defined as mRS > 2 at the final follow-up. Secondary outcomes were residual headache, visual deficit (any persistent decrease in visual acuity or visual field defect related to CVT), late seizure (defined as any seizure during the follow-up period), and neurologic deficit (any language or motor impairment) at the final follow-up. Residual headache was defined as a frequent headache attack (more than once a week) or severe headache (requiring bed rest or hospital admission) after CVT and during the recent 3 months. For patients who were lost to follow-up, the outcome at discharge was considered the final outcome.

We analyzed the impact of anticoagulation delay on outcomes among the entire cohort and in the subgroups with isolated intracranial hypertension, any parenchymal lesion on admission neuroimaging, and coma.

### Statistical analysis

Continuous variables were expressed as the mean ± standard deviation (SD) or median (IQR), as appropriate, and were checked for normal distribution using the Kolmogorov-Smirnov test. Categorical variables were expressed as percentages. Bivariate analyses using the t-test or Mann-Whitney U test for continuous variables and chi-square test for categorical variables were performed to compare baseline variables and outcomes between patients in the earlier and later anticoagulation groups. Imbalanced baseline variables (p < 0.1 in a bivariate analysis) and recognized prognostic factors were entered into a multivariable logistic regression analysis, which included anticoagulation delay as the independent variable, mRS > 2 at final follow-up as the dependent variable, and gender, age, cancer, central nervous system infection, mental disturbance, coma, cerebral hemorrhage, and deep venous system thrombosis as covariates [1]. Statistical analysis was performed using SPSS 23.0 (IBM Corp). All tests were 2-tailed, and p values < 0.05 were considered significant.

## Supplementary Material

Supplementary Table
