# Supplementary material for: Anticoagulation delay does not affect the functional outcome of cerebral venous thrombosis
Source: Aging (Albany NY). 2020 Jun 18;12(12):11835–42. doi: 10.18632/aging.103353 (PMC7343482; doi:10.18632/aging.103353)
Supplement: Supplementary Table [file aging-12-103353-s001..pdf]

SUPPLEMENTARY TABLE

Supplementary Table 1. Univariate data.

Variables	OR	95% CI	p value
Gender	0.602	0.237-1.529	0.286
Age	1.017	0.986-1.049	0.277
Cancer	1.550	0.165-14.583	0.702
CNS infection	3.111	0.863-11.220	0.083
Mental disturbance	3.821	1.430-10.210	0.008
Coma	8.643	2.643-28.262	< 0.001
Cerebral hemorrhage	4.493	1.713-11.782	0.002
Deep venous system thrombosis	1.592	0.526-4.821	0.411
Anticoagulation delay	0.881	0.350-2.219	0.788

Abbreviations: OR, odds ratio; CI, confidence interval; CNS, central nervous system.
